# Association between Red Cell Distribution Width and Outcomes of Nonagenarians Admitted to the Intensive Care Unit—A Retrospective Cohort Study

**DOI:** 10.3390/diagnostics13203279

**Published:** 2023-10-23

**Authors:** Pauline Theile, Jakob Müller, Rikus Daniels, Stefan Kluge, Kevin Roedl

**Affiliations:** 1 Department of Intensive Care Medicine, University Medical Centre Hamburg-Eppendorf, 20246 Hamburg, Germany; pline.theile@gmail.com (P.T.); jmueller@tabea-krankenhaus.de (J.M.); hh_rikus@hotmail.de (R.D.); s.kluge@uke.de (S.K.); 2 Department of Anaesthesiology, Tabea Hospital, 22587 Hamburg, Germany

**Keywords:** nonagenarians, very elderly, red cell distribution width, RDW, critical illness, ICU, critically ill

## Abstract

The red cell distribution width (RDW) measures the heterogeneity of the erythrocyte volume. Different clinical conditions are associated with increased RDW, and high levels (>14.5%) have been described as a predictive marker for unfavorable outcomes and mortality in critically ill patients. However, there is a lack of data on very elderly critically ill patients. Therefore, we aimed to investigate the association of RDW with outcomes in critically ill patients ≥ 90 years. A retrospective analysis was conducted for all consecutive critically ill patients ≥ 90 years who were admitted to the Department of Intensive Care Medicine of the Medical University Centre Hamburg-Eppendorf (Hamburg, Germany) with available RDW on admission. Clinical course and laboratory were analyzed for all patients with eligible RDW. High RDW was defined as (>14.5%). We clinically assessed factors associated with mortality. Univariable and multivariable Cox regression analysis was performed to determine the prognostic impact of RDW on 28-day mortality. During a 12-year period, we identified 863 critically ill patients ≥ 90 years old with valid RDW values and complete clinical data. In total, 32% (*n* = 275) died within 28 days, and 68% (*n* = 579) survived for 28 days. Median RDW levels on ICU admission were significantly higher in non-survivors compared with survivors (15.6% vs. 14.8%, *p* < 0.001). Overall, 38% (*n* = 327) had low, and 62% (*n* = 536) had high RDW. The proportion of high RDW (>14.5%) was significantly higher in non-survivors (73% vs. 57%, *p* < 0.001). Patients with low RDW presented with a lower Charlson Comorbidity Index (*p* = 0.014), and their severity of illness on admission was lower (SAPS II: 35 vs. 38 points, *p* < 0.001). In total, 32% (*n* = 104) in the low and 35% (*n* = 190) in the high RDW group were mechanically ventilated (*p* = 0.273). The use of vasopressors (35% vs. 49%, *p* < 0.001) and renal replacement therapy (1% vs. 5%, *p* = 0.007) was significantly higher in the high RDW group. Cox regression analysis demonstrated that high RDW was significantly associated with 28-day mortality [crude HR 1.768, 95% CI (1.355–2.305); *p* < 0.001]. This association remained significant after adjusting for multiple confounders [adjusted HR 1.372, 95% CI (1.045–1.802); *p* = 0.023]. High RDW was significantly associated with mortality in critically ill patients ≥ 90 years. RDW is a useful simple parameter for risk stratification and may aid guidance for the therapy in very elderly critically ill patients.

## 1. Introduction

Decreasing fertility and increased life expectancy are currently leading to the rapid aging of the world’s population. The population of very old patients is also expected to grow substantially; it is expected that there will be more than 30 million people who are over 90 years old by 2030 [1]. Accordingly, in addition to demographic development, medical processes, and opportunities lead to a growing proportion of very elderly patients requiring emergency and intensive care treatment [2]. To date, about 15% of critically ill patients in the intensive care unit (ICU) have an age of more than 80 years, and 1% are over 90 years [3,4]. Although acceptable outcomes among very elderly critically ill patients have been observed, discussions about the limitations of therapy and futility are ongoing and controversial [4,5]. Common laboratory parameters and clinical scores can be used to support decisions regarding further therapy.

The red cell distribution width (RDW) is an easily measured laboratory test. It is routinely presented in complete blood count profiles. Generally, the RDW is a marker that measures the heterogeneity of erythrocyte volumes (anisocytosis) and assesses variation in the size and form of erythrocytes. Of interest, several clinical conditions in critically ill and non-critically ill patients are associated with increased RDW [6,7,8,9]. High levels, mainly described as RDW values > 14.5%, were described as a predictive marker for unfavorable outcomes and mortality [6,7,8,9]. This association was also shown in elderly patients who were not critically ill [10,11]. Investigations in elderly, especially very old patients, admitted to the ICU addressing the usefulness and predictive ability of RDW compared to other ICU-specific scores are scarce.

Therefore, with the current investigation, we sought to test the hypothesis of whether higher levels of RDW are associated with mortality in an unselected cohort of nonagenarians admitted to an ICU of a large tertiary care university hospital.

## 2. Materials and Methods

### 2.1. Study Design, Setting and Ethics

Data of all adult patients ≥ 90 years consecutively admitted to the Department of Intensive Care Medicine at the University Medical Centre Hamburg-Eppendorf (Hamburg, Germany) between January 2008 and April 2019 were analyzed. The department consists of 12 intensive care units (ICU) and cares for all critically ill adult patients of the hospital, with a total capacity of 140 beds. The Ethics Committee of the Hamburg Chamber of Physicians was informed about the study (No.: 2022-300219-WF). Due to the retrospective nature of the study, the need for informed consent was waived.

### 2.2. Inclusion and Exclusion Criteria

All consecutive adult patients (≥90 years) admitted to the ICU with a valid value of red cell distribution width on admission were included in the study. All patients < 90 years of age, patients with incomplete clinical data, missing admission lab or without the measurement of red cell distribution width were excluded.

### 2.3. Data Collection

Data were collected through an electronic patient data management system (PDMS, Integrated Care Manager^®^ (ICM), Version 9.1—Draeger Medical, Luebeck, Germany). The extracted data included age, gender, comorbidities, admission diagnosis, length of ICU and hospital stay, outcome, treatment modalities and organ support (mechanical ventilation, vasopressor, renal replacement therapy, blood transfusions, antibiotics, antivirals, etc.) and laboratory parameters. A routine laboratory assessment was performed on a daily basis within clinical routines, following local standard operating procedures.

### 2.4. Study Definitions and Patient Management

We defined an RDW value of >14.5% as high and ≤14.5% as low as previously reported [7].

The severity of illness was evaluated via a sequential organ failure assessment (SOFA) [12] and simplified acute physiology score (SAPS II) [13] on admission. The Charlson Comorbidity Index (CCI) [14] was calculated in all patients. Sepsis and septic shock were defined according to the 2016 Third International Consensus Definition for Sepsis and Septic Shock [15].

### 2.5. Statistical Analysis

Data are presented as absolute numbers and the relative frequency or median with an interquartile range (IQR). Categorial variables were compared via Chi-square analysis or Fisher’s exact test, as appropriate. Continuous variables were compared via the Mann–Whitney U-Test. We clinically assessed factors associated with mortality. Univariable and multivariable Cox regression analysis was performed to determine the prognostic impact of RDW on 28-day mortality with associations expressed as hazard ratios (HR) and 95% confidence intervals (CI). The diagnostic test accuracy of RDW and other ICU scores (SAPS II, SOFA) was assessed by receiver operating characteristics (ROC) expressed as their area under the curve (AUROC). Statistical analysis was conducted using IBM SPSS Statistics Version 24.0 (IBM Corp., Armonk, NY, USA). Generally, a *p*-value < 0.05 was considered statistically significant. This study was prepared in accordance with the STROBE (strengthening the reporting of observational studies in Epidemiology) recommendations [16].

## 3. Results

### 3.1. Study Population and Baseline Characteristics

**Figure 1:**
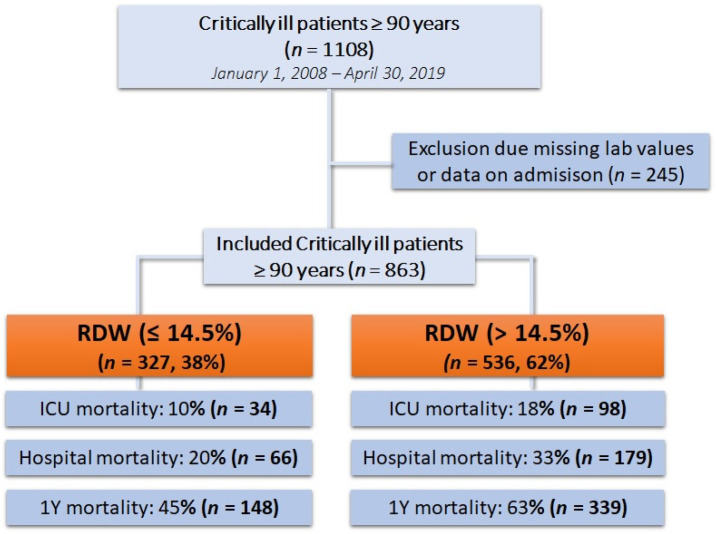
Flow chart of the study.

During the study period from 1 January 2008 to 30 April 2019, we identified 1108 critically ill patients ≥ 90 years. After the exclusion of patients with missing lab values or missing clinical data on admission, we included 863 patients in the final analysis (see study flow chart—Figure 1). The baseline characteristics of the study population are shown in Table 1.

### 3.2. Clinical Characteristics and Laboratory Findings Stratified by Survival Status after 28 Days

Of the 863 included patients, 32% (*n* = 275) died within 28 days, and 68% (*n* = 579) survived for 28 days; nine patients were lost to follow-up after hospital discharge. The median RDW levels on ICU admission were significantly higher in non-survivors compared with survivors (15.6% vs. 14.8%, *p* < 0.001). Also, the proportion of high RDW (>14.5%) was significantly higher in non-survivors (73% vs. 57%, *p* < 0.001). Non-survivors were more often male (34% vs. 32%, *p* = 0.484) and had a lower BMI (22.9 vs. 23.8; *p* = 0.107). Admission to the ICU was significantly more often due to the primary medical cause in non-survivors compared with survivors (46% vs. 26%, *p* < 0.001). The severity of illness on admission to the ICU was significantly higher in non-survivors (SAPS II: 46 vs. 33 points, *p* < 0.001 and SOFA: 4 vs. 1 point, *p* < 0.001). Comorbidities represented by the Charlson Comorbidity Index were higher in non-survivors (1 vs. 1 point, *p* = 0.003). Non-survivors more often received mechanical ventilation (57% vs. 23%, *p* < 0.001), needed more vasopressor therapy (67% vs. 33%, *p* < 0.001) and renal replacement therapy (6% vs. 2%, *p* = 0.002). Non-survivors presented with lower hemoglobin levels, a higher leukocyte count, c-reactive protein, creatinine and LDH levels on admission. Anemia, according to WHO criteria and severe anemia, was present in 84% (*n* = 231) and 11% (*n* = 29) of non-survivors, as well as 85% (*n* = 493) and 5% (*n* = 29) of survivors. Further detailed laboratory and blood gas analyses are reported in Table 2.

### 3.3. Clinical Characteristics and Laboratory Findings Stratified by High or Low RDW

Of the whole study cohort, we identified 38% (*n* = 327) with a low RDW (≤14.5%) and 62% (*n* = 536) with a high RDW (>14.5%). Detailed demographic, clinical characteristics and laboratory values are reported in Table 3. Age, gender and median BMI, as well as the cause of admission to the ICU, were distributed equally in the low and high RDW groups. Patients with a low RDW presented with a lower CCI (*p* = 0.014). Detailed information regarding comorbidities can be found in Supplementary Table S1. The severity of illness on admission was lower in patients in the low RDW group (SAPS II: 35 vs. 38 points, *p* < 0.001; SOFA: 2 vs. 3 points, *p* < 0.001). In total, 32% (*n* = 104) in the low and 35% (*n* = 190) in the high RDW group were mechanically ventilated (*p* = 0.273). The use of vasopressors (35% vs. 49%, *p* < 0.001) and renal replacement therapy (1% vs. 5%, *p* = 0.007) was significantly higher in the high RDW group. Patients with higher RDW had significantly lower hemoglobin and higher bilirubin with the C-reactive protein as well as creatinine. The duration of ICU and hospital stay was a median of 1.4 and 10.2 days in the low RDW group compared with 1.8 and 11.6 days in the high RDW group, respectively. Mortality after 28 days was 23% (*n* = 75) and 37% (*n* = 200), and mortality after 90 days was 31% (*n* = 102) and 48% (*n* = 259) in patients in the low and high RDW group, respectively (both *p* < 0.001). See also Kaplan–Meier survival estimates Supplementary Figure S1. 

### 3.4. Association of RDW with 28-Day Mortality

**Figure 2:**
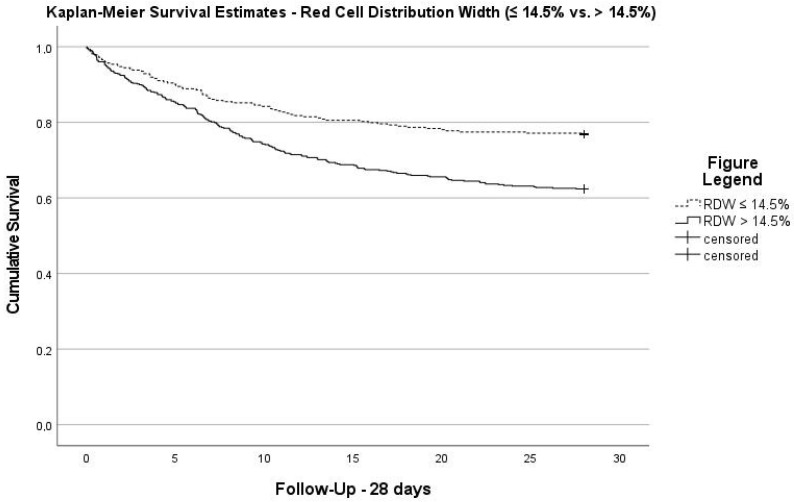
28 d-Kaplan–Meier survival estimates stratified according to red cell distribution width (≤14.5% vs. >14.5% (Log-Rank: *p* < 0.001).

The univariable Cox regression analysis demonstrated that RDW, as a categorical variable at a cut-off level of 14.5%, was significantly associated with 28-day mortality [crude HR 1.768, 95% CI (1.355–2.305); *p* < 0.001]. After multivariable adjustment for gender, SAPS II, myocardial infarction, chronic lung disease, chronic kidney disease and diabetes mellitus, mechanical ventilation and vasopressor therapy, the association remained significant [adjusted HR 1.372, 95% CI (1.045–1.802); *p* = 0.023] (see Supplementary Table S2). Kaplan–Meier estimates showed a significantly higher risk of 28-day mortality with high RDW (log-rank: *p* < 0.001; see Figure 2). The AUROC for the prediction of 28-day mortality of RDW on admission was 0.65 and increased to 0.71 when using the highest RDW during the first 72 h. Other scores, such as SAPS II, presented an AUROC of 0.75 and SOFA of 0.69.

## 4. Discussion

In this study, we investigated the prognostic impact of high RDW levels on admission for mortality in a large cohort of critically ill patients ≥ 90 years admitted to the ICU. We found that RDW on admission was significantly associated with short- and long-term mortality in very elderly critically ill patients. Further, high RDW levels on admission were observed in patients with a higher comorbidity burden and higher severity of illness. Furthermore, RDW was found to be an independent predictor of mortality. To our knowledge, this is the first study to investigate the impact of RDW in a very elderly critically ill cohort.

The red cell distribution width is a very easily accessible laboratory marker, which is calculated automatically within the workup of the complete blood count. A variety of different causes leads to ineffective erythropoiesis and pathological volume distribution. In general, RDW expresses the heterogeneity of erythrocyte volumes. Therefore, the variation in size and form of erythrocytes can be assessed. In recent years, the value of RDW as a prognostic marker for unfavorable outcomes and mortality has been described in different medical conditions, mainly in a non-critically ill setting [10,11]. However, in the critically ill setting (e.g., cardiac arrest, sepsis, …) RDW was also found to be associated with an unfavorable outcome. High levels, mainly described as RDW values > 14.5%, were found to be useful as a predictive marker for mortality [6,7,8,9]. It has been shown that RDW can be useful in elderly patients [17]. But to our knowledge, there are no data on the use and predictive capability of RDW in very elderly critically ill patients. In particular, the prognostic potential of RDW is of interest in the cohort of very elderly patients because it is routinely included in the automated complete blood count.

In general, the mechanisms linking RDW to adverse patient outcomes remain incompletely understood and involve different pathways, including chronic inflammation, chronic diseases, malnutrition, iron deficiency and B12 or folate deficiency [18]. Furthermore, factors that appear in the intensive care unit, like microangiopathic changes, DIC, or pre-existing valvular heart disease, have a relevant influence on RDW levels. However, it has been hypothesized that RDW may reflect the patient’s degree of physiological reserve [18,19]. Especially in the very elderly age group, frailty, which is also linked to physiological reserve, is increasingly recognized to be a factor for unfavorable outcomes [20,21]. As we did not measure frailty in this cohort, we are unable to link this to our study, but this should be evaluated in future studies on this specific age group.

In the current study, we observed that the absolute RDW level, as well as the proportion of high RDW, was significantly higher in non-survivors compared to survivors. Non-survivors in our cohort presented with a higher severity of illness. Accordingly, these patients had a higher need for organ support. They also had lower hemoglobin levels and a higher rate of severe anemia, which is in line with an earlier study [22]. In addition to RDW, we found that other laboratory parameters (e.g., CRP, bilirubin, leukocytes, etc.) were significantly different in survivors and non-survivors. Of interest, one large study in critically ill patients showed that the association of RDW with ICU mortality was strongest in younger patients than in older patients [18]. However, the association of RDW with mortality also remains statistically significant in older patients. This can also be shown in elderly patients admitted to the emergency department [23]. However, it has also been shown that the complete blood count in healthy individuals changes during the course of time. For RDW, it is proposed that the RDW level increases at approximately 1% per annum above the age of 60 years [24]. In the current study, we observed that about two-thirds presented with an elevated RDW. Whether this is an effect of age in the patients per se or the critical illness needs to be clarified. The current analysis of very elderly patients showed a significant association between RDW and mortality. In particular, RDW also served as an independent predictor of mortality in the current cohort during the Cox regression analysis. Furthermore, we also observed that RDW was not only capable of predicting short-term outcomes but also long-term outcomes in very elderly critically ill patients. This is in line with earlier studies, which also showed this in an unselected cohort of critically ill patients [18].

As RDW is routinely included in the automated complete blood count, it is a predictive marker with easy access. Specifically, early prognostication could be of high interest in the very elderly patient cohort. Within our cohort, we observed that RDW showed good AUC discrimination, and this was even higher when we used the highest RDW within the first 72 h of admission to the ICU. However, compared to other established scoring systems like SAPS II and SOFA, RDW showed lower predictive discrimination. We do not expect that a single parameter can outperform the complex score, but it could be used to identify patients at risk of mortality and give an early and quick assessment of the severity of an illness. Furthermore, it may be used as a factor in clinical triage decisions. However, decisions regarding the further care of very elderly patients should not be based on one marker alone. Instead, decisions regarding critical and end-of-life care have to be in accordance with patient wishes. Further, pre-morbid status, as well as the patient’s current medical situation has to be taken into account. Also, the quality of life in the elderly plays a very important role; a critical illness can lower the quality of life considerably, and, therefore, this should also be discussed. In addition to established scoring systems and patient wishes, easily accessibly markers, like RDW, can be of significant importance in guiding patient therapy and decision making. Additionally, it should be noted that RDW is routinely measured and, thus, is free of additional costs.

This study has several limitations. First, we reported from a retrospective study with its common limitations. Second, we reported from a single-center study; thus, the results could be influenced by local clinical practice and incidence of comorbidities and may not be transferable to other settings. Third, the average RDW on admission was 15.0%, which is slightly higher than that reported in the literature. This is probably related to only focusing on RDW at admission, and we are not able to describe its clinical role in the further ICU course. However, we focused on the admission and early prognostic role of RDW. Fourth, changes in clinical practice over time may have influenced the RDW admission levels and outcomes of critically ill elderly patients. Fifth, practice regarding end-of-life care in very elderly patients was not monitored and could have changed.

## 5. Conclusions

In conclusion, this study demonstrates that RDW on admission is strongly associated with short- and long-term mortality in critically ill, very elderly patients. Furthermore, high RDW levels were able to detect patients at risk for mortality. Therefore, RDW levels on admission could be used to easily identify patients at risk of death and may aid guidance for further clinical management and support decision making in very elderly, critically ill patients.

## Figures and Tables

**Table 1:** Baseline characteristics of the study population.

*Variables*	*All* (*n* = 863)
Age (years)	92.2 (90.0–94.0)
Males	279 (32)
Weight (kg)	65 (56–74)
Height (cm)	165 (160–170)
BMI (kg/m^2^)	23.4 (21.0–26.0)
***Primary Admission***	
- Medical	280 (33)
- Surgical—planned	313 (36)
- Surgical—emergency	264 (31)
***Disease Severity***	
SAPS II—admission (pts.)	36 (28–46)
SOFA—admission (pts.)	2 (1–5)
***Comorbidities***	
Charlson Comorb. Index, pts.	1 (0–2)
Arterial hypertension (*n*, %)	609 (71)
Chronic kidney disease (*n*, %)	199 (23)
Coronary heart disease (*n*, %)	127 (15)
Congestive heart failure (*n*, %)	176 (20)
Diabetes mellitus (*n*, %)	115 (13)
Chronic lung disease (*n*, %)	69 (8)
Anemia (WHO criteria) (*n*, %)	731 (85)
Severe anemia (<8 g/dL) (*n*, %)	58 (7)
***Malignancy***	
Solid tumor (*n*, %)	81 (9)
Leukemia (*n*, %)	5 (1)
Lymphoma (*n*, %)	8 (1)
Solid tumor with metastases (*n*, %)	30 (3)
***Outcome***	
Duration ICU stay (days)	1.7 (1.0–3.8)
Duration hospital stay (days)	11.1 (7.3–16.7)
Died in ICU (*n*, %)	132 (15)
Died in hospital (*n*, %)	245 (28)
28 d mortality (*n*, %)	275 (32)
90 d mortality (*n*, %)	361 (42)

Data are expressed as *n* (%) or median (interquartile range); Abbreviations: kg, kilogram; m, meter; ICU, intensive care unit; BMI, body mass index; pts, points.

**Table 2:** ICU Characteristics of patients stratified according to 28-day survival status. (*From the total cohort (n = 863)—9 patients were lost to follow-up*).

*Variables*	*28-Day—Non-Survivor* *(n = 275)*	*28-Day—Survivor* *(n = 579)*	*p*-Value
Age (years)	92.4 (91.1–94.2)	92.1 (90.1–93.9)	0.09
Males (*n*, %)	94 (34)	184 (32)	0.484
BMI (kg/m^2^)	22.9 (21.0–25.7)	23.9 (21.1–26.4)	0.107
***Primary Admission***			
- Medical (*n*, %)	126 (46)	150 (26)	<0.001
- Surgical—planned (*n*, %)	60 (22)	250 (43)	<0.001
- Surgical—emergency (*n*, %)	85 (31)	177 (31)	0.92
***Disease Severity***			
Charlson Comorb. Index, pts.	1 (1–3)	1 (0–2)	0.003
SAPS II—admission (pts.)	46 (36–56)	33 (26–40)	<0.001
SOFA—admission (pts.)	4 (2–8)	1 (0–4)	<0.001
SOFA—24 h (pts.)	5 (2–9)	1 (0–3)	<0.001
***Respiratory support***			
Invasive MV (*n*, %)	158 (57)	134 (23)	<0.001
Duration of MV (days)	1.0 (0.4–2.4)	0.3 (0.1–0.9)	<0.001
***Procedures/Therapies***			
Vasopressors (*n*, %)	184 (67)	191 (33)	<0.001
Renal replacement therapy (*n*, %)	17 (6)	12 (2)	0.002
Hypoxic Liver Injury (*n*, %)	28 (10)	6 (1)	<0.001
Jaundice > 3 mg/dL (*n*, %)	9 (3)	3 (1)	0.001
***Laboratory results***			
Hemoglobin—admission	10.1 (9.1–11.5)	10.3 (9.3–11.6)	0.054
Anemia (WHO criteria) (*n*, %)	231 (84)	493 (85)	0.337
Severe anemia (<8 g/dL) (*n*, %) RDW—admission	29 (11)	29 (5)	0.352
RDW > 14.5%—admission	15.6 (14.5–17.1)	14.8 (13.9–15.8)	<0.001
MCV—admission	200 (73)	330 (57)	<0.001
Leukocytes—admission	91 (87–96)	91 (87–94)	0.063
Thrombocytes—admission	11.8 (8.9–15.8)	10.2 (7.6–13.6)	<0.001
LDH—admission	210 (159–298)	211 (159–272)	0.718
Bilirubin—admission (mg/dL)	281 (220–442)	241 (200–297)	<0.001
CRP—admission	0.7 (0.5–1.0)	0.6 (0.4–0.8)	0.003
Creatinine—admission	39 (10–100)	25 (6–72)	<0.001
	1.2 (0.9–1.9)	1.0 (0.8–1.4)	<0.001
***Blood gas analysis***			
Lactate, mmol/L—admission	1.5 (1.0–2.5)	1.0 (0.7–1.3)	<0.001
pH, level—admission	7.36 (7.31–7.41)	7.38 (734–7.42)	<0.001
Base excess—admission	−2 (−6–1)	−0.3 (−29–2.1)	<0.001
Bicarbonate—admission	23 (20–25)	24 (22–26)	<0.001
paO_2_—admission	101 (78–142)	92 (74–123)	0.030
paCO_2_—admission	41 (35–47)	41 (37–46)	0.460
pH—nadir	7.34 (7.23–7.44)	7.38 (7.33–7.44)	<0.001
Lactate—peak	2.4 (1.6–4.5)	1.5 (1.2–2.2)	<0.001
***Outcome***			
Duration ICU stay (days)	2.7 (1.1–6.0)	1.4 (0.9–2.9)	<0.001
Duration hospital stay (days)	8.8 (4.2–13.8)	12.0 (8.4–17.5)	<0.001

Data are expressed as n (%) or median (interquartile range), Abbreviations: ICU, intensive care unit; BMI, body mass index; kg, kilogram; m, meter; pts, points; LDH, lactate-dehydrogenase; CRP, c-reactive protein; RDW, red cell distribution width; MV, mechanical ventilation; SAPS II, simplified acute physiology score II; SOFA, sequential failure organ assessment.

**Table 3:** ICU Characteristics of patients stratified according to red cell distribution width (≤14.5% vs. >14.5%).

*Variables*	*RDW ≤ 14.5%* *(n = 327)*	*RDW > 14.5%* *(n = 536)*	*p*-Value
Age (years)	92.1 (90.9–93.8)	92.3 (90.9–94.1)	0.302
Males (*n*, %)	112 (34)	167 (31)	0.346
BMI (kg/m^2^)	23.4 (21.3–26.4)	23.4 (20.8–25.9)	0.35
***Primary Admission***			
- Medical (*n*, %)	100 (31)	180 (34)	0.361
- Surgical—planned (*n*, %)	122 (37)	191 (36)	0.62
- Surgical—emergency (*n*, %)	104 (32)	160 (30)	0.546
***Disease Severity***			
Charlson Comorb. Index, pts.	1 (0–2)	1 (1–3)	0.014
SAPS II—admission (pts.)	35 (26–43)	38 (29–48)	<0.001
SOFA—admission (pts.)	2 (0–4)	3 (1–6)	<0.001
SOFA—24 h (pts.)	1 (0–4)	3 (1–5)	<0.001
***Respiratory support***			
Invasive MV (*n*, %)	104 (32)	190 (35)	0.273
Duration of MV (days)	0.5 (0.2–1.2)	0.7 (0.2–1.8)	0.249
***Procedures/Therapies***			
Vasopressors (*n*, %)	114 (35)	264 (49)	<0.001
Renal replacement therapy (*n*, %)	4 (1)	25 (5)	0.007
Hypoxic Liver Injury (*n*, %)	10 (3)	24 (4)	0.298
Jaundice > 3 mg/dL (*n*, %)	5 (2)	7 (1)	0.786
***Laboratory results***			
Hemoglobin—admission	10.9 (9.8–12.1)	10.0 (8.8–11.2)	<0.001
RDW—admission	13.8 (13.4–14.2)	15.9 (15.2–17.2)	<0.001
MCV—admission	91 (88–95)	90 (86–94)	<0.001
Leukocytes—admission	10.5 (7.7–13.5)	10.9 (8.0–14.8)	0.096
Thrombocytes—admission	202 (162–256)	217 (154–295)	0.057
LDH—admission	248 (202–305)	255 (216–354)	0.101
Bilirubin—admission (mg/dL)	0.6 (0.4–0.8)	0.6 (0.5–1.0)	0.032
CRP—admission	21 (5–63)	38 (10–86)	<0.001
Creatinine—admission	1.0 (0.8–1.4)	1.1 (0.8–1.7)	<0.001
***Blood gas analysis***			
Lactate, mmol/L—admission	1 (0.8–1.5)	1.1 (0.8–1.9)	0.039
pH, level—admission	7.39 (7.34–7.43)	7.37 (7.32–7.41)	0.001
Base excess—admission	0 (−3–2)	−1 (−5–2)	0.020
Bicarbonate—admission	24 (22–26)	24 (21–25)	0.024
paO_2_—admission	94 (76–132)	94 (74–129)	0.865
paCO_2_—admission	40 (36–46)	41 (37–45)	0.186
pH—nadir	7.38 (7.33–7.45)	7.37 (7.31–7.43)	0.024
Lactate—peak	1.6 (1.2–2.5)	1.8 (0.7–2.8)	0.173
***Outcome***			
Duration ICU stay (days)	1.4 (0.9–3.2)	1.8 (0.9–3.9)	0.026
Duration hospital stay (days)	10.2 (7.3–14.1)	11.6 (7.4–18.6)	0.004
28 d mortality (*n*, %)	75 (23)	200 (37)	<0.001
90 d mortality (*n*, %)	102 (31)	259 (48)	<0.001

Data are expressed as n (%) or median (interquartile range); Abbreviations: ICU, intensive care unit; BMI, body mass index; kg, kilogram; m, meter; pts, points; LDH, lactate-dehydrogenase; CRP, c-reactive protein; RDW, red cell distribution width; MV, mechanical ventilation; SAPS II, simplified acute physiology score II; SOFA, sequential failure organ assessment.

## Data Availability

The datasets supporting the conclusions of this article are included within the article.

## Supplementary Materials

The following supporting information can be downloaded at: https://www.mdpi.com/article/10.3390/diagnostics13203279/s1, Supplementary Table S1: Pre-existing comorbidities; Supplementary Table S2: Cox regression model for RDW and factors associated with 28 d mortality; Supplementary Figure S1: 1-year Kaplan–Meier survival estimates stratified according red cell distribution width (≤14.5% vs. >14.5%).
