# Supplementary material for: Association between Red Cell Distribution Width and Outcomes of Nonagenarians Admitted to the Intensive Care Unit—A Retrospective Cohort Study
Source: Diagnostics (Basel). 2023 Oct 23;13(20):3279. doi: 10.3390/diagnostics13203279 (PMC10605993; doi:10.3390/diagnostics13203279)
Supplement: Supplementary file 1 [file diagnostics-13-03279-s001.zip › diagnostics-2653710-supplementary.pdf]

Supplementary Figure S1: 1-Year Kaplan-Meier survival estimates stratified according Red Cell Distribution Width (≤ 14.5% vs. > 14.5%) (Log-Rank: $p < 0.001$)

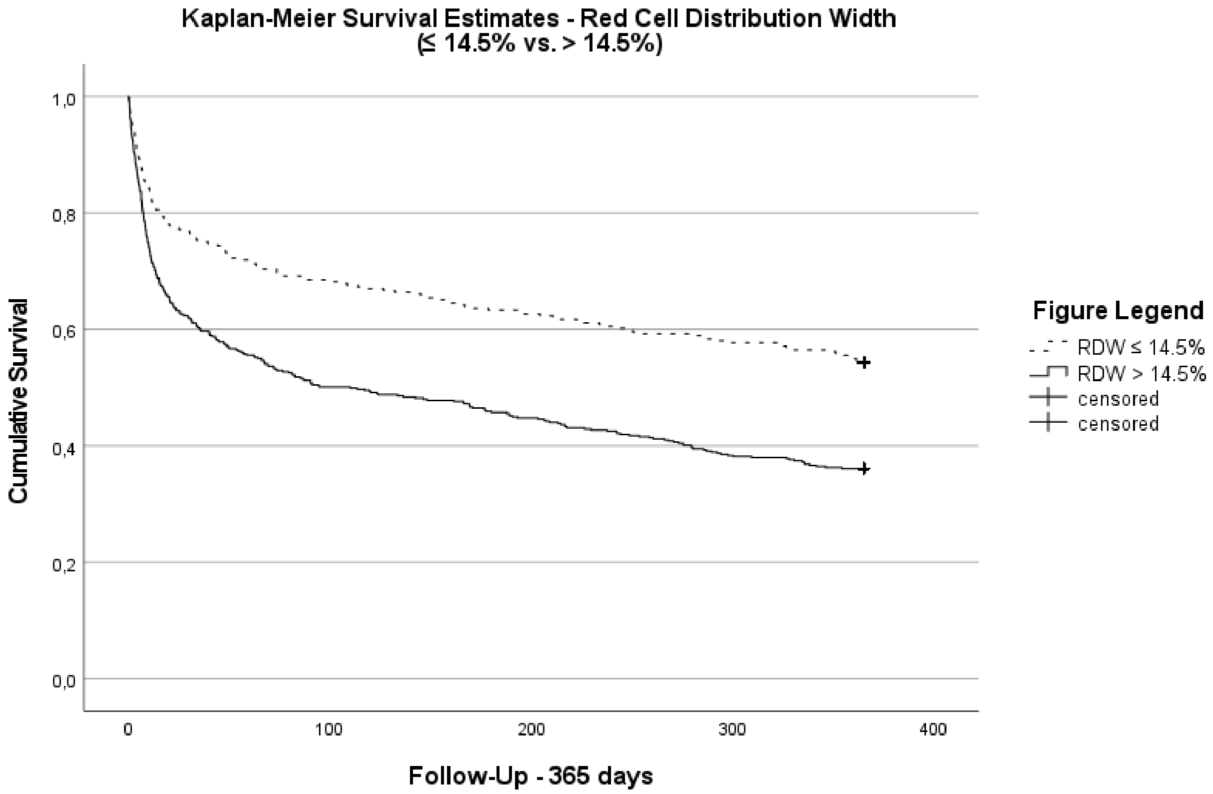

Supplementary Table S1 – Pre-existing comorbidities

Parameters	***RDW ≤ 14.5%*** *(n = 327)*	***RDW > 14.5%*** *(n = 536)*	*p*-value
**AIDS** *n (%)*	0 (0)	1 (0)	0.434
**Cerebral arterial disease** *n (%)*	49 (15)	90 (17)	0.484
**Chronic lung disease** *n (%)*	23 (7)	46 (9)	0.416
**Chronic kidney disease** *n (%)*	56 (17)	143 (27)	0.001
**Congestive heart disease** *n (%)*	50 (15)	126 (24)	0.004
**Connective tissue disease** *n (%)*	2 (1)	0 (0)	0.070
**Coronary heart disease** *n (%)*	56 (17)	71 (13)	0.119
**Dementia** *n (%)*	57 (17)	110 (21)	0.265
**Diabetes Mellitus** *n (%)*	46 (14)	69 (13)	0.617
**Liver cirrhosis** *n (%)*	3 (1)	4 (1)	0.786
**Peripheral arterial disease** *n (%)*	27 (9)	51 (10)	0.532
**Malignancy** *n (%)*			
Solid tumor	28 (9)	53 (10)	0.517
Leukemia	1 (0)	4 (1)	0.408
Lymphoma	1 (0)	7 (1)	0.137
Solid tumor with metastases	9 (3)	21 (4)	0.364

Abbreviations: AIDS, acquired immune deficiency syndrome; n, number; RDW, red cell distribution width;

Supplementary Table S2 – Cox-Regression model for RDW and factors associated with 28d-Mortality

Cox regression	**Covariables**	HR (95% CI)	*p* value
Final Model	**RDW** (high vs. low)	1.372 (1.045 – 1.802)	0.023
	**Gender** (male vs. female)	1.007 (0.782 – 1.297)	0.955
	**SAPS II** (points)	1.051 (1.040 – 1.061)	< 0.001
	**Myocardial Infarction** (yes vs. no)	1.164 (0.836 – 1.622)	0.368
	**Chronic Lung Disease** (yes vs. no)	0.752 (0.477 – 1.184)	0.218
	**Chronic kidney disease** (yes vs. no)	0.979 (0.744 – 1.289)	0.881
	**Diabetes Mellitus Type II** (yes vs. no)	0.744 (0.504 – 1.097)	0.136
	**Mechanical Ventilation** (yes vs. no)	1.568 (1.169 – 2.103)	0.003
	**Vasopressors** (yes vs. no)	1.513 (1.113 – 2.058)	0.008

Abbreviations: HR, hazard ratio; CI, confidence interval; RDW, red cell distribution width; SAPS II, simplified acute physiology score II;
